# Ultra-low-field MRI: a David versus Goliath challenge in modern imaging

**DOI:** 10.1007/s11547-025-02091-y

**Published:** 2025-09-26

**Authors:** Cesare Gagliardo, Paola Feraco, Eleonora Contrino, Costanza D’Angelo, Laura Geraci, Giuseppe Salvaggio, Andrea Gagliardo, Ludovico La Grutta, Massimo Midiri, Maurizio Marrale

**Affiliations:** 1 Department of Biomedicine, Neurosciences and Advanced Diagnostics (BIND), University of Palermo, Via del Vespro, 129, 90127 Palermo, Italy; 2 Neuroradiology Unit, University-Hospital Paolo Giaccone, Palermo, Italy; 3 Centre for Medical Sciences (CISMed), University of Trento, Trento, Italy; 4 Department of Health Promotion, Mother and Child Care, Internal Medicine and Medical Specialties (ProMISE), University of Palermo, Palermo, Italy; 5 Department of Precision Medicine in the Medical, Surgical, and Critical Care Area (MePreCC), University of Palermo, Palermo, Italy; 6 Neuroradiology Unit, Department of Radiological Sciences, A.R.N.A.S. Civico, Palermo, Italy; 7 Radiology Unit, University-Hospital Paolo Giaccone, Palermo, Italy; 8 Clinical Neurophysiology Unit, Sleep Lab, “Clinical Course”, Palermo, Italy; 9 Department of Physics and Chemistry “Emilio Segrè”, University of Palermo, Palermo, Italy

**Keywords:** Magnetic resonance imaging (MRI), Ultra-low-field MRI (ULF-MRI), Portable MRI (pMRI), Point-of-care systems, Neuroimaging, Innovation

## Abstract

Ultra-low-field magnetic resonance imaging (ULF-MRI), operating below 0.2 Tesla, is gaining renewed interest as a re-emerging diagnostic modality in a field dominated by high- and ultra-high-field systems. Recent advances in magnet design, RF coils, pulse sequences, and AI-based reconstruction have significantly enhanced image quality, mitigating traditional limitations such as low signal- and contrast-to-noise ratio and reduced spatial resolution. ULF-MRI offers distinct advantages: reduced susceptibility artifacts, safer imaging in patients with metallic implants, low power consumption, and true portability for point-of-care use. This narrative review synthesizes the physical foundations, technological advances, and emerging clinical applications of ULF-MRI. A focused literature search across PubMed, Scopus, IEEE Xplore, and Google Scholar was conducted up to August 11, 2025, using combined keywords targeting hardware, software, and clinical domains. Inclusion emphasized scientific rigor and thematic relevance. A comparative analysis with other imaging modalities highlights the specific niche ULF-MRI occupies within the broader diagnostic landscape. Future directions and challenges for clinical translation are explored. In a world increasingly polarized between the push for ultra-high-field excellence and the need for accessible imaging, ULF-MRI embodies a modern “*David versus Goliath*” theme, offering a sustainable, democratizing force capable of expanding MRI access to anyone, anywhere.

## Introduction

Magnetic resonance imaging (MRI) is a cornerstone in medicine and in neuroscience, offering unparalleled soft-tissue contrast and spatial resolution essential for diagnosing and managing central nervous system diseases without exposing patients to ionizing radiation. Most clinical MRI systems today operate at magnetic fields between 1.5 and 3 Tesla, with research centers increasingly exploring ultra-high-field (≥ 7 Tesla) imaging. However, high-field MRI systems come with notable limitations, such as high installation costs, the need for dedicated infrastructure, and safety concerns for certain patient populations (i.e., those with implants) [1–5]. Additionally, concerns regarding environmental sustainability have arisen, particularly due to the significant energy consumption and reliance on helium, a limited and costly resource [6–11]. Recent developments in superconducting magnet technologies aim to reduce helium usage and promote greener, more sustainable imaging solutions [12]. Moreover, MRI has experienced a growing role as an imaging tool for guiding therapeutic procedures, including interventional radiology and image-guided treatments, enabling precise navigation and real-time feedback during minimally invasive interventions [13–19].

In the modern era, MRI has thus evolved into a versatile, multiparametric, and multiplanar imaging modality underpinning critical decisions in the diagnostic and therapeutic pathways of numerous patients [20]. This aligns with the current medical trend toward personalized and precision medicine, where diagnostic imaging directly informs tailored patient care. Furthermore, the emergence of radiomics and theranostics, which integrates diagnostics and targeted therapy, further amplifies MRI’s potential to guide highly individualized treatments [21–24].

In contrast, ultra‐low-field MRI (ULF-MRI) systems, typically operating below 0.2 Tesla, present an attractive alternative in scenarios where portability, lower cost, or patient safety is paramount. The resurgence of interest in ULF-MRI has been driven by technological advances in magnet and radiofrequency (RF) coil design [25, 26], pulse sequence optimization [27–31], and sophisticated image reconstruction methods [28]. Recent prototypes have demonstrated that portable devices can produce diagnostically useful images despite inherent limitations in SNR and image resolution [32–41]. Such systems offer the promise of bedside imaging in emergency departments, intensive care units, operating rooms and resource-limited settings [42].

This narrative review provides an in-depth overview of ULF-MRI. It begins by briefly summarizing the fundamental physical concepts underlying ULF-MRI, followed by a discussion of the technical challenges associated with low-field imaging. Subsequent sections detail recent hardware and software innovations that have improved image quality, followed by a review of clinical applications where ULF-MRI shows promise with a comparison with other imaging modalities, including low-field, high-field, ultra-high-field MRI, and computerized tomography (CT). Finally, this review addresses future directions and the challenges that must be overcome to further integrate ULF-MRI into clinical practice.

## Research methodology

This narrative review was based on a structured literature search designed to capture the current state of knowledge on ULF-MRI and its emerging role in clinical imaging, starting also from earlier articles to reconstruct the evolution that has led to the current state of the field. A systematic search of electronic databases (including PubMed, Scopus, IEEE Xplore, and Google Scholar) was conducted up to August 11, 2025. Search strategies employed a combination of Medical Subject Headings (MeSH) and free-text keywords such as *“ultra-low-field MRI”*, *“low-field MRI”*, *“portable MRI”*, *“point-of-care imaging”*, *“magnet design”*, *“RF coil development”*, *“pulse sequence optimization”*, *“image reconstruction algorithms”*, “*denoising*,” “*super-resolution*,” and *“AI-assisted MRI reconstruction”*. Boolean operators (AND, OR) were used to refine search outputs and ensure both breadth and specificity.

To provide a contextual and comparative framework, the search also included literature on related imaging technologies and adjacent topics. Specific terms, such as *“computed tomography”*, “CT,” *“photon-counting CT”, “PCCT”*, *“sustainable imaging”*, *“low-resource imaging environments”*, and *“MRI accessibility”*, were used to identify sources relevant to the broader narrative of diagnostic democratization and technological contrasts. Gray literature (i.e., conference abstracts, technical white papers, preprints, and reports from regulatory agencies or manufacturers) was included when peer-reviewed data were unavailable or insufficient, especially in areas concerning hardware innovation and emerging clinical use cases.

Articles were selected based on originality, scientific rigor, and relevance to one or more core themes: physics and engineering of ULF-MRI systems (including magnet topology, RF coil design, and low-field-specific pulse sequences), software developments (notably in AI-based denoising and reconstruction), and clinical deployment scenarios. Reference lists of selected papers and recent reviews were manually screened to identify additional relevant sources. Only English-language publications were included. The final synthesis aimed to integrate foundational concepts, recent technical breakthroughs, and translational applications into a coherent narrative reflecting the potential and limitations of ULF-MRI within modern imaging, also including earlier works that help elucidate the pathway leading to the current state of the field.

## Physical principles and technical considerations

### Basic physics of ultra‐low-field MRI

At its core, MRI relies on the phenomenon of nuclear magnetic resonance (NMR), wherein the hydrogen nuclei (protons) in water and fat align with an external static magnetic field (B₀). The net magnetization (M₀) produced in tissue is directly proportional to the strength of B₀ [43]. Consequently, lower fields in ULF-MRI lead to reduced magnetization and inherently lower SNR. The Larmor frequency, given by ω₀ = γB₀ (with γ representing the gyromagnetic ratio), is also decreased at ultra‐low fields, which affects both the efficiency of RF excitation and signal detection.

Despite these challenges, several advantages arise at ultra‐low fields. For instance, the lower Larmor frequencies result in reduced RF energy deposition in tissues, leading to lower specific absorption rates (SAR) [3]. This makes ULF-MRI inherently safer for patients with metallic implants, for those admitted to intensive care units (ICUs) who require continuous monitoring or connection to medical devices that are not necessarily MRI-compatible, and for individuals with other contraindications to conventional MRI. Additionally, at lower magnetic field strengths, magnetic susceptibility effects, which can lead to artifacts near tissue interfaces, are minimized [44]. These features make ULF-MRI a particularly compelling option for imaging in complex clinical scenarios where the use of conventional high-field MRI may present significant challenges.

### Challenges related to signal-to-noise ratio (SNR), contrast-to-noise ratio (CNR), and acquisition time

**Fig. 1:**
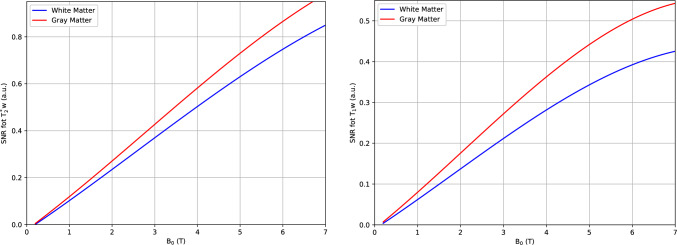
Signal-to-noise ratio for T_2_^*^-weighted (left) and T_1_-weighted (right) acquisitions in gray matter (red line) and white matter (blue line) as a function of magnetic field strength (up to 7 T). Both gray and white matter exhibit a progressive increase in SNR values with increase in magnetic field strength (a.u., arbitrary units; T_2_^*^w, T_2_^*^-weighted; T_1_w, T_1_-weighted). Plotted values are derived from literature [45, 50]

**Fig. 2:**
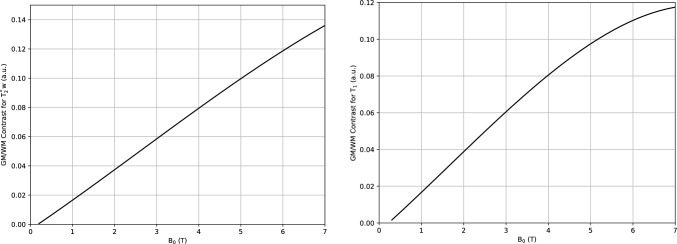
Contrast between gray matter (GM) and white matter (WM) for T_2_^*^-weighted (left) and T_1_-weighted acquisitions as a function of magnetic field strength (up to 7 T). Both acquisitions exhibit a progressive increase of the GM/WM contrast with increase in magnetic field strength. Plotted values are derived from Marques et al. [50]

A central challenge in ULF-MRI is the reduced SNR and CNR compared with higher-field systems, as both parameters generally scale positively with the static magnetic field strength (B₀). The MRI signal is proportional to the induced nuclear magnetization, which increases linearly with the strength of the static magnetic field B₀, and to the rate of change of magnetic flux according to Faraday’s law. This rate corresponds to the signal detected at the magnetization’s precession frequency and likewise increases linearly with B_0_ [45–49]. Empirical and theoretical models indicate that SNR is approximately proportional to a power of the field strength, often described as B₀^3,2^ [45], though actual scaling factors can be lower depending on the contrast mechanism and tissue properties (Fig. 1). Similarly, CNR generally increases with B₀, although its scaling is sequence- and tissue-dependent, reflecting variations in intrinsic relaxation times and susceptibility contrast (Fig. 2). This revised understanding suggests that low-field MRI may suffer a smaller SNR and CNR penalty than previously assumed, with implications for the perceived performance gap between ultra-low and high-field systems. In conventional MRI, higher B_0_ values yield greater net magnetization and, thus, stronger signals, which in turn contribute to high SNR, improved CNR and enhanced spatial resolution [50]. Noise in MRI originates from several sources, including thermal noise within the patient and electronic noise from the detection system [45, 51–53]. In ULF-MRI, the reduction in signal is generally more significant, although some sources of noise such as thermal noise are also reduced due to the lower induced currents [3].

As the magnetic field decreases, SNR and CNR efficiency reduce. To maintain image quality (specifically, consistent SNR and CNR) at lower field strengths, it is necessary to either increase the acquisition time or decrease the spatial resolution. The acquisition time *T*_ACQ_ increases supra-linearly with decreasing magnetic field and follows:

$$T_{{{\text{ACQ}},L}} = \left( {\frac{{B_{0,H} }}{{B_{0,L} }}} \right)^{{2 \cdot {\text{powereff}}}} T_{{{\text{ACQ}},H}}$$

where *T*_ACQ,L_ is the acquisition duration at low field (*B*_0,*L*_), *T*_ACQ,H_ is the acquisition duration at high field (*B*_0,H_), powereff is the effective power law coefficient which depends on image contrast (i.e., ~ 1.04 for ${T}_{2}^{*}$-weighted and ~ 0.90 for *T*_1_-weighted images) [45]. This relationship indicates that doubling the intensity of the magnetic field results in an approximately fourfold reduction in the acquisition time.

Alternatively, spatial resolution can be adjusted based on the following expression illustrating the scaling of spatial resolution between high-field (*B*_0,H_) and low-field (*B*_0,L_) MRI systems [45]:

$${\text{res}}_{L} = \left( {\frac{{B_{0,H} }}{{B_{0,L} }}} \right)^{{{\text{powereff}}/3}} {\text{res}}_{H} .$$

Thus, the achievable resolution at low field (res_*L*_) is derived from the resolution at high field (res_*H*_) by applying a scaling factor based on the ratio of the magnetic field strengths, raised to the power-efficiency coefficient (powereff) divided by three. However, studies show that the drop in image quality at lower fields is not as dramatic as once thought, especially for brain imaging (Marques et al. [45] and references therein). The efficiency of contrast decreases more gradually than expected. In practice, when working with low-field MRI, a compromise is usually made: scan times are slightly increased, and resolution is slightly reduced, so the overall image remains usable without making the scan too long or the image too blurry.

Nevertheless, several strategies have been pursued to counteract the SNR deficit [54] and typical lower image resolution of ULF-MRI [55]. First of all advanced multi-channel phased-array coils design and coils conforming to patient anatomy can improve the filling factor and enhance sensitivity [56]. Novel or optimized pulse sequences, such as ultrashort echo time (UTE) and zero echo time (ZTE) imaging, are designed to capture signal more efficiently and maximize SNR despite the lower magnetization [57]. Techniques, like parallel imaging (i.e., SENSE, GRAPPA) and compressed sensing, help compensate for reduced SNR by enabling shorter acquisition times and improving image quality through advanced signal processing [58, 59].

### Hardware design considerations

ULF-MRI benefits from simpler hardware designs compared to high-field systems. In many cases, permanent magnets or electromagnets capable of generating fields below 0.2 Tesla are sufficient for imaging, and they often require less power and simpler cooling systems than superconducting magnets [45]. This has paved the way for the development of portable and point‐of‐care MRI (pMRI) devices [60]. The design of gradient and RF coils also requires special attention at ultra‐low fields. Gradient coils must be engineered to produce linear magnetic field gradients even at low-field strengths, while RF coils are optimized for the lower Larmor frequencies encountered in ULF-MRI. Advances in coil geometry, impedance matching, interference shielding, and preamplifier technology have been instrumental in enhancing the performance of ultra-low-field systems, helping to mitigate some of the challenges posed by reduced SNR [54, 60, 61].

In addition, some experimental prototype ULF-MRI systems leverage superconducting quantum interference devices (SQUIDs) as highly sensitive detectors (see Table 1), capable of operating at extremely low fields where conventional RF coils would otherwise be ineffective, thus further expanding the potential of ultra-low-field imaging [25, 25, 62, 63].

**Table 1:** Comparison between conventional MRI and SQUID-based ULF-MRI systems

Characteristic	Conventional clinical MRI	SQUID-based ULF-MRI
Sensor type	RF coils	SQUID sensors
Magnetic field strength	High (≥ 1.5 T) or very high (≥ 7 T)	Very low (micro or millitesla)
NMR signal strength	Strong	Weak
Current clinical application	Standard in hospitals	Research, specific niches
Brain signal detection	Limited (via BOLD/fMRI)	High temporal resolution (via MEG)
Specific absorption rates	Higher (especially for high duty-cycle sequences)	Near-zero (inherently safe)

Conventional MRI relies on strong magnetic fields and RF coils to detect NMR signals with relatively high signal-to-noise ratios. In contrast, SQUID-based ULF-MRI systems operate at much lower magnetic fields and use superconducting sensors to detect extremely weak magnetic signals. This makes them particularly valuable in research settings and in other applications of SQUID technology, such as magnetoencephalography (MEG) [64]

## Advances in ultra‐low-field MRI technology

### Magnet and gradient innovations

Recent advances in magnetic materials and magnet engineering have significantly influenced the evolution of ULF-MRI. Modern permanent magnet assemblies, particularly those employing Halbach array configurations [65], are capable of producing homogenous magnetic fields in the 0.05–0.2 Tesla range [54]. Halbach arrays, with their ability to focus the magnetic field in a specific region, are particularly well suited for low-cost or portable systems [66]. These magnet designs not only reduce power consumption and installation complexity but also contribute to lower operational costs [67, 68]. Neodymium–iron–boron (NdFeB) magnets are known for their high magnetic strength and are commonly used in various applications, including medical imaging devices as ULF-MRI systems [69].

The refinement of gradient coil designs has also played an essential role. Newer gradient systems provide improved linearity and temporal stability, which are crucial for executing advanced pulse sequences that require rapid gradient switching and precise spatial encoding [70]. Such improvements have enabled more accurate imaging even at lower magnetic fields, as it is now possible in clinical settings to tailor gradient design to specific applications (i.e., extremity or brain [71]), thereby allowing for a reduction in scanner size, cost, and power consumption [54, 60].

### RF coil and preamplifier developments

Overcoming the inherent low signal levels of ULF-MRI requires innovative RF coil designs [45]. Researchers have developed multi-channel coil arrays that conform to the patient’s anatomy, thereby increasing the filling factor and improving SNR. These coils are often custom-designed for specific imaging applications, allowing for enhanced performance in targeted clinical scenarios [72]. In addition to coil design, advances in preamplifier technology have been pivotal. Cryogenic and other low-noise electronics have been integrated into ULF-MRI systems to minimize electronic noise, helping to offset the reduction in intrinsic signal. These improvements in the RF detection chain are critical for achieving acceptable image quality in low-field systems [72, 73].

### Pulse sequence optimization and reconstruction techniques

Pulse sequence development is another key area of progress for ULF-MRI. Conventional sequences, such as spin echo and gradient echo, have been modified to account for the shorter T₁ relaxation times typical of low-field imaging. Novel sequences, like UTE and ZTE imaging [57], have emerged as effective means to capture rapidly decaying signals at ultra‐low fields, thereby improving the diagnostic yield.

**Fig. 3:**
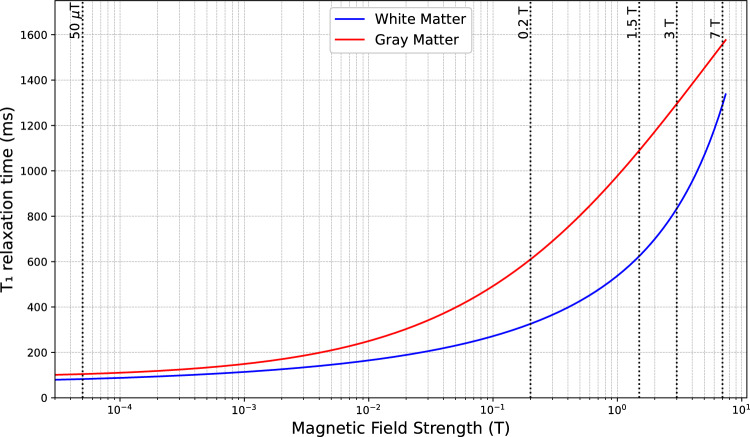
T_1_ relaxation times of gray matter (red line) and white matter (blue line) are shown as a function of magnetic field strength, plotted on a logarithmic scale. The data illustrate the characteristic increase in T_1_ values with rising magnetic field intensity, with a pronounced variation observed at ultra-low-field strengths. This trend highlights the potential for enhanced T_1_-based tissue discrimination in ultra-low-field MRI. This plot was produced by using data and parameters reported by Fisher et al. [75]

**Fig. 4:**
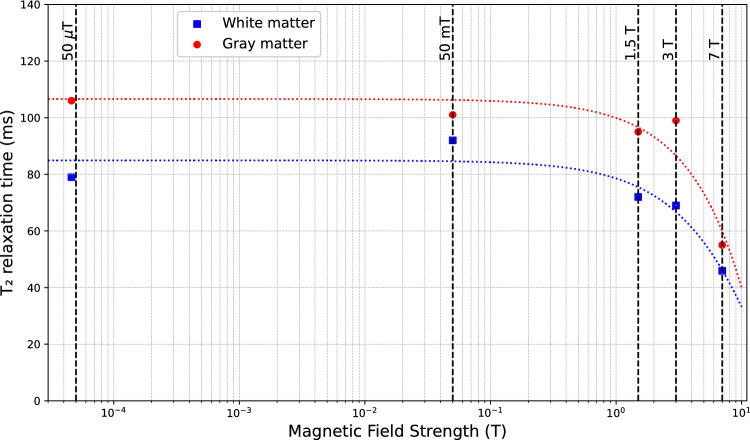
T₂ relaxation times of gray matter (circles; red dotted line indicates the relative trend) and white matter (blue circles; blue dotted line indicates the relative trend) as a function of magnetic field strength (0.05 T, 0.5 T, 1 T, 3 T, 7 T). Both gray and white matter exhibit a progressive decrease in T₂ values with increase in magnetic field strength. This trend reflects enhanced magnetic susceptibility effects and increased spin–spin interactions at higher fields, which have important implications for optimizing T₂-weighted contrast in MRI protocols across various magnetic field strengths. The plotted values were derived from the literature (Volegov et al. [76], O’Reilly et al. [77], Staniz et al. [78] and Uludag et al. [79])

**Fig. 5:**
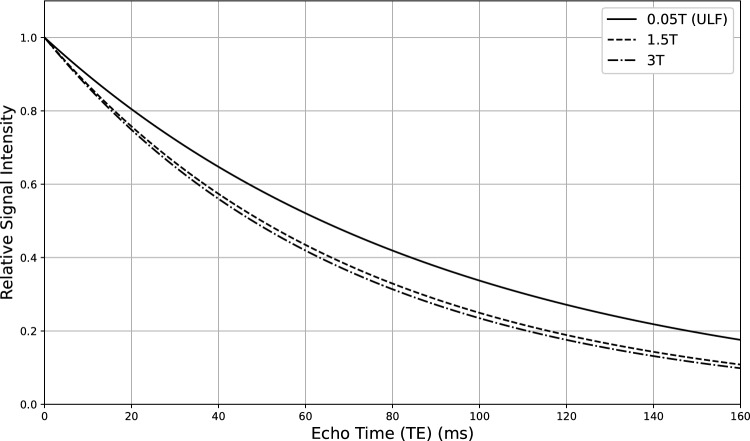
Relative signal intensity decay as a function of echo time (TE) at different magnetic field strengths (0.05 T, 1.5 T, and 3 T). At lower field strengths (0.05 T; solid line), the longer T_2_ relaxation times result in slower signal decay. In contrast, at higher-field strengths (dashed line for 1.5 T and dash-dot line for 3 T), the faster decay reflects shorter T_2_ values. These trends illustrate the field-dependent nature of transverse relaxation and underscore the importance of adjusting T₂-weighted imaging parameters according to the magnetic field regime. Curves reconstructed by using T_2_ values for 0.05 T (O'Reilly et al. [77]) and 1.5 and 3 T (Stanisz et al. [78])

To support the understanding of field-dependent relaxation behavior and to serve as a refresher on the relationship between relaxation times and magnetic field strength, Figs. 3, 4 and 5 summarize how T₁ and T₂ relaxation parameters vary with magnetic field intensity and highlight their implications for image contrast in MRI. Figure 3 shows the increase in T₁ values for gray and white matter across a range of field strengths, with marked changes at ultra-low fields. Figure 4 depicts the progressive decrease in T₂ values with increase in field strength. Figure 5 demonstrates the corresponding variation in signal intensity decay as a function of echo time (TE).

Nevertheless, several hardware- and software-based strategies have been explored to enhance SNR efficiency in ULF-MRI, ranging from optimized k-space sampling and reconstruction to AI-driven denoising, super-resolution, and electromagnetic interference cancelation, each with distinct advantages and limitations depending on the intended application and specific B₀ characteristics [74].

Advanced reconstruction techniques, including parallel imaging methods (i.e., SENSE, GRAPPA) and compressed sensing algorithms [59], have also been applied to ULF-MRI data [28]. These approaches recover high-quality images from undersampled acquisitions, mitigating low SNR and enabling shorter scan times. The integration of these reconstruction strategies has been essential for transitioning ULF-MRI from experimental prototypes to clinically viable systems nowadays available.

A recent study demonstrated the feasibility of magnetization transfer (MT) imaging on a shielding-free 0.055 T ULF-MRI scanner, achieving enhanced contrast with low SAR. Using sinc-modulated RF pulse trains in 3D GRE and FSE sequences, clear MT effects were seen in both phantom and in vivo brain imaging. MT preparation improved tissue contrast and lesion visibility in T_2_-weighted and FLAIR-like images, while SAR levels remained well below FDA limits, supporting ULF-MRI’s potential for safe, low-cost neuroimaging [80].

To address lower SNR, a super-resolution technique was introduced, generating high-resolution isotropic T_2_-weighted images from multiple low-resolution anisotropic scans at 64 mT on a Hyperfine Swoop system. This method achieved 1.5 mm isotropic resolution and consistent T_2_ values, enhancing anatomical detail without extending scan time [81].

Another study proposed a deep learning–based super-resolution framework for brain imaging at 0.055 T. Using dual-acquisition 3D modeling and cross-scale feature extraction, high-quality synthetic T_1_- and T_2_-weighted images were produced in under 20 min, restoring fine anatomical detail with strong agreement to 3 T MRI [82].

Donnay et al. introduced a novel Fourier-based super resolution (FouSR) method, combining orthogonal T_2_-weighted FLAIR acquisitions to enhance image and lesion sharpness without deep learning, offering a practical solution for improving ULF-MRI performance, especially in resource-limited settings [83].

Finally, several preliminary and non-preliminary studies have demonstrated that the application of artificial intelligence-based denoising algorithms is effective in enhancing image quality and improving segmentation accuracy [84–88], and some of these approaches have already been implemented in clinically approved ULF-MRI scanners [89].

### Portable and point‐of‐care systems

One of the most promising developments in ULF-MRI is the emergence of portable systems. Prototypes of transportable MRI scanners operating at fields as low as 0.064 Tesla have demonstrated the feasibility of bringing MRI directly to the patient [32, 33, 35, 40, 49, 71, 90, 91]. Such systems are designed for use in emergency departments, intensive care units, and remote or resource-limited settings, where the logistics and risks of transporting critically ill patients to a fixed imaging suite are prohibitive.

The portability of ULF-MRI not only reduces infrastructural requirements and costs but also expands the reach of diagnostic imaging. In particular, the ability to perform bedside imaging in acute stroke [49, 92], traumatic brain injury [93], or other critical conditions represents a significant advancement in patient care [35, 35, 37, 38, 41, 46, 55, 90, 94–103].

However, it is important to note that portability does not equate to full versatility. ULF-MRI systems typically weigh over 600 kg [104], making them unsuitable for certain types of transport, such as helicopter evacuation. This weight limitation restricts their use in scenarios requiring rapid or airborne deployment (i.e., mobile stroke units in areas that are difficult to reach except by air).

## Clinical applications of ultra‐low-field MRI

### Neuroimaging

Neuroimaging represents one of the most promising applications of ULF-MRI, albeit currently restricted to specific contexts or niches due to many limitations (i.e., lack of advanced imaging capabilities, as well as the need for further validation studies to confirm the reliability and effectiveness of ULF-MRI for broader implementation in new medical settings) as previously reported [105]. Notwithstanding that high-field MRI is the gold standard for brain imaging, ultra-low-field systems have shown considerable potential in detecting gross intracranial pathologies such as hemorrhage [106], edema, and mass lesions [107]. One of the notable advantages of ULF-MRI in neuroimaging is the reduced susceptibility artifacts, which are especially problematic in regions adjacent to the skull base or near metallic implants [47]. This reduction in artifacts enhances the diagnostic clarity in complex anatomical regions although it must contend with lower spatial resolution and reduced SNR. The safety profile of ULF-MRI further contributes to its appeal in neuroimaging. Patients with implanted devices, such as deep brain stimulators or pacemakers, are at higher risk during high-field MRI examinations due to RF-induced heating and other interactions [2]. The lower magnetic field strength and reduced SAR in ULF-MRI allow for safer imaging of these patients, expanding the scope of neuroimaging applications to populations that may otherwise be excluded or get limited access to MRI facilities.

Recent experimental studies have shown that ultra-low-field (0.05 T) time of flight MR-angiography of the head and neck is feasible using simplified scanners without RF or magnetic shielding, with optimized 2D/3D flow-compensated GRE sequences providing sufficient contrast to visualize major intracranial and cervical vessels, including arterial bifurcations [101].

In 30 patients with longstanding rheumatoid arthritis, 0.04 T MRI provided diagnostic evaluation of craniocervical junction lesions, and, when combined with radiography, yielded valuable information for treatment planning, supporting the potential utility of ultra-low-field imaging in chronic cervical spine involvement [108].

After early work on glioma characterization in 1989 using ULF-MRI [27], its role has shifted toward intra- and perioperative applications. With high-field MRI now established as the gold standard for brain tumor assessment, ULF-MRI has been reevaluated for surgical navigation and resection control. After a preliminary case series [102], a study using a mobile 0.15 T MRI scanner (PoleStar N-20) in 63 patients with WHO Grade II–IV gliomas showed that intraoperative ULF-MRI enabled additional tumor removal in up to 47.6% of cases and improved complete resection rates for enhancing lesions (71.2% vs. 52.4%) [103]. These results support ULF-MRI as a useful intraoperative tool, particularly for maximizing resection in selected glioma cases.

In 2012, ULF-MRI was successfully used during transsphenoidal resection of a pituitary macroadenoma in a standard operating room in a low-income country, detecting residual tumor with accuracy comparable to high-field MRI [36]. This first documented case supports its potential to expand neurosurgical capabilities in resource-limited settings, with subsequent studies confirming feasibility for intraoperative sellar region procedures [94–96, 99].

In 204 neurosurgical procedures for glioblastoma (GBM) and cerebral metastases (CM), intraoperative ULF-MRI enabled precise intraoperative localization with neuronavigation, leading to further resection in 20.6% of GBM cases but none for CMs [98]. In cystic metastases, it supported stereotactic aspiration, reducing mass effect and facilitating radiotherapy, suggesting potential utility for surgical guidance and control [98].

ULF-MRI provides reliable measurements of ventricular size in pediatric hydrocephalus, demonstrating strong concordance with standard imaging modalities. Although not suitable as a standalone diagnostic tool, ULF-MRI represents a promising adjunct for the radiological assessment and management of hydrocephalus [109].

Recent evidence highlights the growing role of ULF-MRI as a rapid, cost-effective, and versatile tool for diagnosis, monitoring, and management of stroke and other cerebrovascular diseases [110].

In mild cognitive impairment and Alzheimer’s disease, optimized ULF-MRI with an open-source machine learning pipeline enabled accurate hippocampal and white matter hyperintensity volumetry, in good agreement with conventional MRI [111]. This is a relevant advance given the projected tripling of cases by 2050 [112]. Accurate brain volumetry at ultra-low-field can also be achieved by combining orthogonal T2-weighted acquisitions [113], while deep learning models reduce systematic deviations between 64 mT and 3 T MRI, improving alignment with high-field standards [114]. These findings are promising for the increasingly widespread investigation of neurodegenerative diseases using AI-based approaches applied to volumetric MRI datasets.

In multiple sclerosis, ULF-MRI was well tolerated, preferred by nearly half of participants, and showed high sensitivity and positive predictive value for detecting white matter lesions, particularly periventricular, when read conservatively by trained radiologists [35, 115]. In progressive multifocal leukoencephalopathy, it achieved moderate-to-high accuracy for T2-FLAIR lesions and focal atrophy, with high specificity and good concordance with high-field MRI, supporting its use for longitudinal monitoring in clinical trials and in severely disabled patients [38]. In optic neuritis, portable ULF-MRI demonstrated moderate sensitivity and high specificity for dissemination in space, and its integration into clinical workflows significantly shortened time from symptom onset to imaging, potentially expediting multiple sclerosis diagnosis where access to conventional MRI is limited [116].

In adults with epilepsy, ULF-MRI detected over two-thirds of lesions seen on 3 T scans, with highest accuracy for tumors and post-traumatic lesions, suggesting potential to improve diagnostic access in resource-limited settings [41]. By the same author, a pilot study demonstrated that portable ultra-low-field MRI enabled rapid bedside postictal imaging, revealing focal diffusion abnormalities in the seizure onset zone and supporting its potential role in the presurgical evaluation of epilepsy [37].

Finally, returning to the persistent challenge of standardizing protocols for multicenter neuroradiological studies, the UNITY Project showed that portable 64 mT ultra-low-field MRI systems can maintain robust image quality across diverse global sites and operational environments, supporting their application in large-scale, multicenter neuroimaging research [117].

One of the main current limitations in the field of neuroradiology remains the uncertain and debated potential of using MRI contrast agents with ultra-low-field scanners. The effectiveness and safety of contrast enhancement at such low magnetic fields are still under investigation, and their clinical utility has yet to be clearly established since the contrast agent effect is significantly smaller at lower field strengths with a shorter primary T_1_ time [118].

### Emergency and critical care neuroimaging

One of the most transformative applications of ULF-MRI is in emergency and critical care, where its portability enables bedside imaging, avoiding the risks and delays of transporting unstable patients. In time-sensitive conditions like stroke or traumatic brain injury, rapid access to imaging can greatly impact outcomes [49, 92, 119]. Recent pilot studies have demonstrated that ULF-MRI can identify major intracranial events, such as hemorrhages [106] and ischemic lesions [49] (even in patients with cardiac implantable electronic device [120]), white matter hyperintensities [121] with sufficient sensitivity to guide urgent therapeutic decisions. The ability to perform such imaging at the point of care has the potential to revolutionize acute stroke management and critical care diagnostics, particularly in settings where conventional high-field MRI is impractical or unavailable or in developing low-income countries or and remote or resource-limited settings.

In a multicenter prospective open-label trial involving three Italian centers, an ULF-MRI system is being evaluated for its diagnostic accuracy in guiding acute stroke treatment decisions, with comparison to conventional neuroimaging across multiple time points, integration of AI-based image analysis, and assessment of feasibility, cost-effectiveness, and potential deployment in mobile stroke units [122].

In a pilot study at a European tertiary stroke center, portable ultra-low-field MRI reliably supported treatment decisions in acute ischemic stroke, detecting most lesions seen on high-field MRI and showing potential for use where conventional MRI is unavailable [123].

In patients with intracranial hemorrhage, ULF-MRI demonstrated high concordance with standard neuroimaging for detecting midline shift, with excellent sensitivity and specificity, and provided prognostic information on poor functional outcome, supporting its role as a feasible bedside tool for mass effect assessment [124].

Portable ULF-MRI has been investigated as a diagnostic tool for acute brain injury (ABI) in ICU settings, particularly in patients undergoing extracorporeal membrane oxygenation (ECMO). In a retrospective study of 17 ICU patients, ULF-MRI and head CT were compared for ABI detection within 24 h. No ABI was observed in eight patients, while nine showed pathology: ULF-MRI identified all ischemic lesions, whereas CT detected only half. Conversely, CT detected both hemorrhages, while ULF-MRI detected only one. These findings suggest ULF-MRI may offer superior sensitivity to ischemic injury, although further optimization could improve hemorrhage detection [33]. A subsequent study confirmed that ULF-MRI can be performed across various ECMO cannulation strategies in specially trained ICU settings [32]. Furthermore, ULF-MRI showed excellent interrater reliability and near-perfect agreement with conventional neuroimaging for assessing ventricular size and diagnosing hydrocephalus, supporting its utility as a point-of-care modality in neurocritical care [125].

In critically ill pediatric patients, ULF-MRI proved to be a safe and feasible bedside imaging modality, with good diagnostic concordance with conventional MRI and CT. Despite limitations in diffusion-weighted imaging quality, ULF-MRI demonstrated high specificity and acceptable sensitivity, supporting its potential utility as a complementary neuroimaging tool in acute pediatric settings [126].

In addition to critical and emergency settings, ULF-MRI is emerging as a promising tool for intraoperative and perioperative imaging. Its portability, compatibility with metallic surgical instruments, and reduced infrastructural demands support its use in the operating room to guide neurosurgical procedures, monitor acute changes, and detect complications without interrupting workflow. Early applications suggest that ULF-MRI can complement standard intraoperative modalities by providing continuous, low-risk imaging for real-time decision-making [36, 94–96, 98, 99, 102, 103, 127, 128].

Moreover, the experience from the recent (December 2019–May 2023) COVID-19 pandemic [129] highlighted the importance of point-of-care imaging solutions that minimize patient transport between hospital departments. In this neither desirable nor foreseeable context, ULF-MRI may offer essential bedside imaging, reducing infection risk, optimizing patient management, and enhancing healthcare worker safety [105].

### Advanced neuroimaging applications

One of the major limitations of ULF-MRI systems is their current inability to implement advanced imaging techniques, such as diffusion tensor imaging (DTI), magnetic resonance spectroscopy (MRS), functional MRI (fMRI), and perfusion-weighted imaging (PWI), which are routinely performed with high-field scanners. However, experimental efforts are underway to extend these capabilities to the ultra-low-field domain as well.

DTI is a powerful technique widely used in high-field MRI to map the diffusion of water molecules and infer microstructural properties of biological tissues, particularly in the brain [130]. DTI at ultra-low magnetic fields remains technically challenging due to low signal-to-noise ratios and limited gradient performance. Nonetheless, preliminary studies have shown that diffusion measurements are feasible at ultra-low field, suggesting potential for structural imaging in portable or resource-limited settings, despite current limitations in resolution and precision compared to high-field systems [119].

Another notable application of ULF-MRI is MRS, specifically through the exploitation of J-coupling effects. J-coupling, or scalar spin–spin coupling, represents a promising avenue for advancing spectroscopic studies using ULF-MRI systems. At such low magnetic fields, the reduced chemical shift dispersion is compensated by the increased relative influence of J-coupling interactions, which can provide rich structural information through characteristic multiplet patterns. This feature has the potential to enhance molecular identification and metabolic profiling in environments where high-field magnets are impractical. Although the clinical translation of ultra-low-field spectroscopic techniques remains in its early stages, and significant technical and sensitivity challenges persist, the exploitation of J-coupling phenomena could pave the way for novel diagnostic applications, particularly in portable or resource-limited settings [131].

Functional MRI at ultra-low magnetic fields is challenged by the absence of conventional BOLD contrast due to negligible magnetic susceptibility effects. Nevertheless, simulations suggest that changes in cerebral blood volume (CBV), exploiting T_1_ differences among brain tissues, could enable functional imaging at ultra-low fields. Preliminary results predict feasible signal-to-noise ratios, supporting the potential for combined MEG-MRI studies in portable and resource-limited environments, although with lower spatial and temporal resolution than high-field fMRI [132].

At present, cerebral perfusion imaging using ULF-MRI remains largely experimental and has not yet achieved clinical applicability. Conventional perfusion techniques, such as dynamic susceptibility contrast (DSC) MRI, arterial spin labeling (ASL), and dynamic contrast-enhanced (DCE) MRI, rely on magnetic susceptibility effects and high signal-to-noise ratios, both of which are substantially diminished at ultra-low field. As a result, traditional methods for assessing cerebral blood flow and volume are not directly transferrable to ultra-low-field environments.

Magnetic resonance fingerprinting (MRF) [133] offers a potential solution to the low SNR and image quality limitations of ULF-MRI. Studies on 50 mT systems using optimized acquisition schedules and microdictionary approaches have shown that MRF can estimate T1, T2, and B_1_ + with good agreement to gold-standard methods, suggesting its potential to enhance diagnostic capability and tissue characterization in portable point-of-care ULF-MRI [134].

### Other applications

Musculoskeletal imaging is another domain where ULF-MRI is gaining traction. Although high-field MRI offers superior spatial resolution, ULF-MRI can be effective for evaluating joint abnormalities, soft-tissue injuries, and inflammatory conditions [47, 135–137]. The lower field strength minimizes artifacts that often compromise image quality in the presence of metal implants, a common scenario in postoperative patients [47, 138]. This characteristic makes ULF-MRI particularly useful for follow-up imaging after orthopedic surgeries.

Additionally, the portability of ULF-MRI systems makes them well suited for outpatient clinics or field settings, where conventional imaging resources may be limited. Although the spatial resolution in ULF-MRI is lower than in high-field systems, the diagnostic information provided is often sufficient for assessing gross musculoskeletal pathology, thereby offering a practical and cost-effective alternative [137, 139].

A recent study introduced a low-cost, whole-body ULF-MRI scanner operating at 0.05 Tesla, designed to improve MRI accessibility in resource-limited settings. The system requires no RF or magnetic shielding, runs on standard power, and integrates deep learning techniques for both electromagnetic interference suppression and image reconstruction. Clinical imaging protocols were implemented across multiple anatomical regions with acceptable scan times (< 8 min), demonstrating the feasibility of high-utility diagnostic imaging despite strong electromagnetic interference. This approach highlights the potential of deep learning-enhanced ULF-MRI as a scalable and patient-friendly solution for global healthcare challenges [140].

A pilot feasibility study is currently evaluating ULF-MRI for breast imaging, focusing on the development of novel pulse sequences and reconstruction algorithms to enhance image quality. The study includes healthy volunteers and breast cancer patients, comparing ULF-MRI with standard modalities such as mammography, ultrasound, conventional MRI, clinical exams, and pathology. It also investigates a potential breast cancer biomarker (intrinsic T_1_rho dispersion) which reflects tissue protein content and water-macromolecule proton exchange, with expected differences between fat, fibroglandular, and tumor tissues [141].

## Comparative analysis of imaging modalities

An essential part of evaluating ULF-MRI involves comparing it with other imaging modalities. Table 2 provides a comparison of ultra‐low-field MRI, low‐field MRI, high‐field MRI, ultra‐high-field MRI, and computed tomography (CT) across several key features. Despite evident limitations (some of which have now been partially resolved or mitigated) ultra-low-field MRI is increasingly gaining a meaningful niche between the current gold-standard first-line imaging modality, CT (due to its speed, cost-effectiveness, and immediate interpretability), and higher-field MRI systems. However, compared to CT, significant limitations persist, particularly in terms of acquisition time. It will be interesting to observe future advancements in spectral and photon-counting CT technologies (PCCT) [142–149], which could further disrupt and redefine this already evolving and somewhat uncertain diagnostic landscape. Regulatory approvals, including FDA/CE marking (already granted for certain ULF-MRI systems [150]), could further accelerate ULF-MRI adoption and integration into clinical practice.

**Table 2:** Comparison of CT and MRI systems by magnetic field strength (specific system performance and clinical suitability will depend on individual hardware design, sequence optimization, and application requirements)

Parameter	CT	Ultra-low-field MRI	Low-field MRI	High-field MRI	Ultra-high-field MRI
Magnetic field strength	N/A (uses X-rays)	< 0.2 Tesla	0.2–1 Tesla	1.5–3 Tesla	≥ 7 Tesla
Gantry shape; length	Circular (donut-shaped); short (~ 20–30 cm)	Open designs; short (~ 50 cm)	Circular or open designs, weight-bearing or standing setups available; long (~ 100–150 cm)	Circular (some 1.5 T systems with open designs available); longer (~ 150–200 cm)	Circular; longer (~ 150–200 cm)
SNR	High spatial resolution; SNR varies by dose	Low (improved by advanced coils/sequences/AI)	Moderate	High	Very high (with challenges in homogeneity)
Image contrast	Excellent for bone; low/moderate for soft tissue	Good for gross pathology; limited fine detail	Good balance; lower detail than high-field	Excellent soft-tissue contrast and resolution	Superior detail; sensitive to subtle variations
Susceptibility artifacts	N/A (artifacts are generally due to beam hardening)	Minimal (due to low-field strength)	Present but reduced compared to high-field	Increased, especially near metal/air interfaces	Pronounced; requires advanced correction techniques
Safety (implants, SAR)	Safe regarding implants; uses ionizing radiation	Very high safety; low SAR; compatible with implants	Generally safe; some limitations in SAR management	Increased RF energy deposition; safety concerns for some implants	Higher risk; requires stringent safety measures
Portability	Portable options exist (i.e., mobile CT units)	High (portable, point‐of‐care systems available but still too heavy, not transportable by helicopter)	Generally fixed; some mobile designs in development	Fixed installations; large and heavy systems	Fixed; typically research installations
Acquisition time	Fast imaging modality; typically seconds; excellent for emergency imaging	Generally, longer due to lower SNR; optimized with recent sequences and AI-based algorithms	Moderate	Shorter acquisition times possible with high SNR	Typically longer due to sequence complexity
Cost and infrastructure	Moderate for MDCT; high for PCCT; MDCT cost-effective for many applications	Low to moderate; reduced installation requirements	Moderate; requires dedicated rooms	High cost; significant infrastructure investment	Very high cost; mostly limited to research centers
Maintenance costs	Low for MDCT; high for PCCT	Low to moderate	Moderate	High	Very High
Clinical applications	Broad use in trauma, oncology, and emergency settings	Neuro, musculoskeletal, emergency/critical care; bedside imaging	Broad applications (also weight-bearing systems) with moderate resolution	Standard clinical applications in most areas	Advanced research and specialized clinical applications
Sustainability	Low (particularly for PCCT, due to high energy consumption)	Very high (no superconducting magnet, low power consumption)	Moderate to high (1.0 T systems use superconducting magnets)	Low (higher for “sealed” systems with low helium requirements, although power consumption remains high)	Very Low

SNR, Signal-to-noise ratio; SAR, specific absorption rate; AI, artificial intelligence; MDCT, multi-detector CT; PCCT, photon-counting CT

## Discussion

Ultra-low-field magnetic resonance imaging is emerging as a versatile diagnostic tool that complements, rather than replaces, conventional imaging modalities. Its key advantages (portability, lower cost, enhanced safety for patients with implants, reduced power consumption, and infrastructure requirements) position ULF-MRI as an attractive solution for specific clinical scenarios, particularly in emergency and critical care settings. Technological advancements, including innovations in magnet design, RF coil engineering, pulse sequence optimization, and AI-driven reconstruction methods, have substantially mitigated some of the traditional limitations associated with low-field imaging, such as reduced SNR, CNR, and lower spatial resolution.

Comparative analysis with other imaging modalities underscores ULF-MRI’s potential to occupy a unique niche between CT, prized for its speed and availability, and high-field MRI, valued for its superior tissue contrast, versatility and advanced imaging capabilities. In neuroimaging, musculoskeletal imaging, and intraoperative use, ULF-MRI has demonstrated clinically relevant diagnostic performance, particularly when immediate imaging is needed at the point of care, with growing evidence supporting additional promising clinical applications in the future. Additionally, the experience of the COVID-19 pandemic has reinforced the importance of minimizing patient transport within healthcare facilities. This highlighting the strategic role ULF-MRI could play in enhancing infection control and workflow efficiency. Although several challenges remain such as limited capabilities for advanced imaging techniques like fMRI, MRS, DTI, and perfusion studies, ongoing technological innovation is likely to expand ULF-MRI’s applications and improve its diagnostic robustness. A further critical limitation is the current inability, at ultra-low field, to exploit T2*-weighted imaging for the reliable detection of cerebral microbleeds and, more broadly, for visualizing paramagnetic blood products. This constraint, primarily due to the inherently low susceptibility contrast at ultra-low-field strengths, significantly hampers the assessment of pathologies where blood degradation products serve as crucial imaging biomarkers. Future research priorities include improving SNR strategies through further coil refinement, noise reduction, and AI integration; further hardware miniaturization to enhance portability; expanding clinical validation through multicenter trials; and exploring hybrid approaches integrating ULF-MRI with other technologies. Furthermore, the increasing emphasis on sustainability principles, ethical healthcare delivery, and accessibility in low-income settings strengthens the case for broader adoption of ULF-MRI, particularly in resource-constrained environments.

It will also be essential for imaging professionals to develop familiarity with ULF-MRI to ensure accurate interpretation and avoid diagnostic pitfalls [151].

Overall, while still evolving, ULF-MRI’s unique combination of portability, safety, and cost-effectiveness positions it as a highly attractive modality for clinical scenarios where conventional high-field MRI may be impractical or contraindicated. In the modern radiology scenario, where the blanket is still inevitably short, having more arrows in the quiver (each suited to different targets) is not a limitation, but may result in a strategic advantage.

## Conclusion

Ultra-low-field MRI is emerging as a transformative technology with the potential to reshape diagnostic imaging. Despite limitations in SNR and spatial resolution, advances in magnet design, RF coil development, pulse sequences, and reconstruction techniques are steadily enhancing its performance. ULF-MRI’s low SAR, reduced susceptibility artifacts, and portability make it particularly suited for emergency, neuro, and musculoskeletal imaging, as well as for patients with implants. While high- and ultra-high-field MRI will remain the gold standards for high-resolution applications, ULF-MRI fills a critical niche by offering a cost-effective, safe, and accessible alternative, especially for bedside imaging and use in low-resource or remote settings. Its lower energy consumption and infrastructural demands also align with green economy principles, making it attractive for sustainable healthcare models and for supplementary research applications. Future advancements in AI integration, hardware miniaturization, and clinical validation will be essential for broader adoption. In this evolving landscape, the story of ULF-MRI echoes a modern “*David versus Goliath*” narrative, where a smaller, adaptable technology challenges the dominance of high-field systems through innovation and strategic advantage. As innovation continues, ULF-MRI is poised to become an increasingly valuable, patient-centered tool within modern diagnostic imaging.
